# Biomedical potential of *Anabaena variabilis* NCCU-441 based Selenium nanoparticles and their comparison with commercial nanoparticles

**DOI:** 10.1038/s41598-021-91738-7

**Published:** 2021-06-29

**Authors:** Bushra Afzal, Durdana Yasin, Haleema Naaz, Neha Sami, Almaz Zaki, Moshahid Alam Rizvi, Raj Kumar, Pooja Srivastava, Tasneem Fatma

**Affiliations:** 1 Department of Biosciences, Jamia Millia Islamia (Central University), New Delhi, 110025 India; 2 Institute of Nuclear Medicine and Allied Sciences (INMAS), Defence Research and Development Organization (DRDO), New Delhi, 110054 India

**Keywords:** Biotechnology, Microbiology, Nanoscience and technology

## Abstract

Selenium nanoparticles (SeNPs) are gaining importance in the field of medicines due to their high surface area and unique properties than their other forms of selenium. In this study, biogenic selenium nanoparticles (B-SeNPs) were synthesized using cyanobacteria and their bioactivities (antioxidant, antimicrobial, anticancer and biocompatibility) were determined for comparison with commercially available chemically synthesized selenium nanoparticles (C-SeNPs). Color change of reaction mixture from sky blue to orange-red indicated the synthesis of biogenic SeNPs (B-SeNPs). UV–Vis spectra of the reaction mixture exhibited peak at 266 nm. During optimization, 30 °C of temperature, 24 h of time and 1:2 concentration ratio of sodium selenite and cell extract represented the best condition for SeNPs synthesis. Various functional groups and biochemical compounds present in the aqueous extract of *Anabaena variabilis* NCCU-441, which may have possibly influenced the reduction process of SeNPs were identified by FT-IR spectrum and GC–MS. The synthesized cyanobacterial SeNPs were orange red in color, spherical in shape, 10.8 nm in size and amorphous in nature. The B-SeNPs showed better anti-oxidant (DPPH, FRAP, SOR and ABTS assays), anti-microbial (antibacterial and antifungal) and anti-cancer activitities along with its biocompatibility in comparison to C-SeNPs suggesting higher probability of their biomedical application.

## Introduction

Selenium nanoparticles (SeNPs) are gaining more attention nowadays because of their high surface area, more surface activity, high adsorbing, catalytic efficiency and biocompatibility^1–4^. SeNPs are usually synthesized by physical (UV radiations, laser ablation, hydrothermal techniques etc.)^5,6^ and chemical (precipitation method, acid decomposition, reduction by using ascorbic acid, sodium dodecyl sulfate, glucose and sulfur dioxide etc.)^7–9^. All these methods use harsh chemicals, high temperature and acidic pH which make the nanoparticles unsafe for biomedical use^3^ whereas biosynthesized SeNPs are inexpensive, eco-friendly and produce no toxic byproducts during their synthesis^3^.

Biological synthesis of SeNPs have been reported from bacteria-*Pseudomonas* sp. and *Paracoccus denitrificans*^10^, *Shewanella oneidensis*^11^, *Klebsiella pneumonia*^12^, *Pseudomonas alcaliphila*^13^, *Zooglea ramera*^14^, *Gliocladium roseum*^15^, *Bacillus* sp.^16^, *Bacillus megaterium*^17^; fungi-*Mariannaea* sp. HJ^18^, *Aspergillus terreus*^19^, *Pleurotus tuber-regium*^20^ and plants-*Diopyros Montana*^21^, *Allium sativum*^22^, *Zingiber officinale*^23^, *Vitis vinifera*^24^, *Psidium guajava*^25^, *Emblica officinali*^26^, *Citrus limon*^27^ etc.

Crude water based extracts of 20 cyanobacterial strains (*Anabaena variabilis* NCCU-441, *Arthrospira indica*, *Arthrospira maxima*, *Arthrospira indica*, *Calothrix brevissima*, *Chroococcus* sp., *Gloeocapsa gelatinosa*, *Lyngbya* sp., *Microchaete* sp., *Nostoc muscorum*, *Nostoc punctiforme*, *Nostoc sphericum*, *Oscillatoria* sp., *Spirulina* sp., *Phormidium* sp., *Plectonema* sp., *Scytonema* sp., *Spirulina platensis*, *Synechocystis* sp., *Westiellopsis prolifica*) have been used recently by us for the screening of their potential to synthesize SeNPs^28^. Polysachcharides from cyanobacteria (*Spirulina plantensis*)^29^ and seaweed (*Undaria pinnatifida*)^30^ have also been used to synthesize selenium nanoparticles.

The aim of the present study was to synthesize biogenic SeNPs (B-SeNPs) using cell free extract of filamentous heterocystous cyanobacteria-*Anabaena variabilis* NCCU-441 and to perform their physicochemical and biological characterization (anti-oxidant, anti-microbial and anti-cancer). The biological activities of biologically synthesized SeNPs (B-SeNPs) were also compared with the commercially available chemically synthesized selenium nanoparticles (C-SeNPs) for their future applications.

## Results

### Optimization and synthesis of B-SeNPs

**Figure 1:**
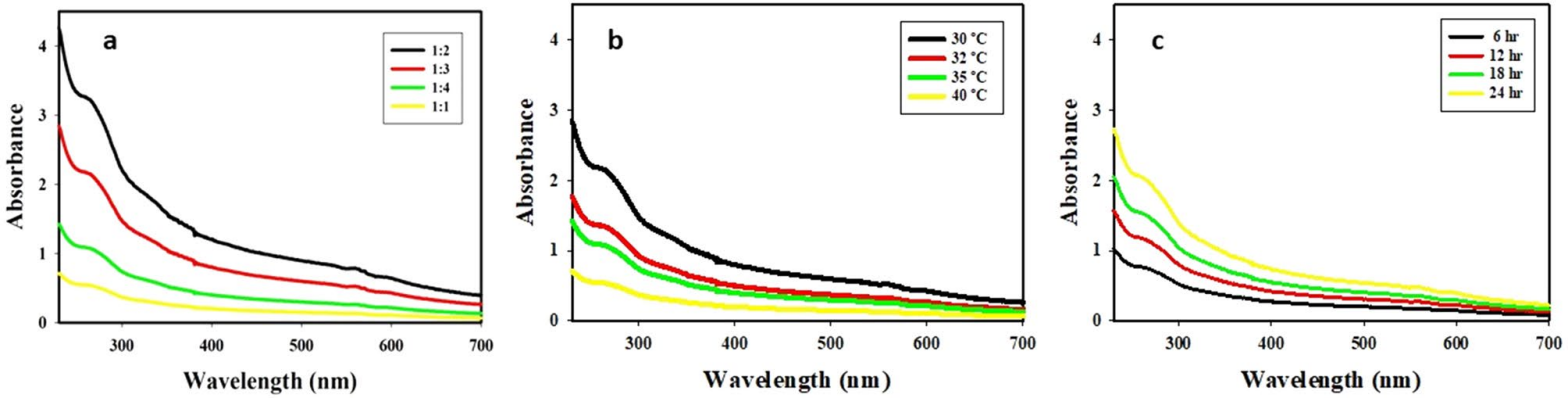
Optimization for B-SeNPs synthesis (**a**) for sodium selenite and cell extract concentration ratio (**b**) for temperature (**c**) reaction time.

**Figure 2:**
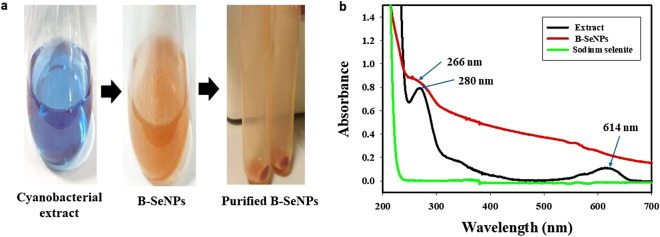
Synthesis of biogenic SeNPs (**a**) visual change in the reaction mixture after SeNPs synthesis, (**b**) UV–Vis spectra of B-SeNPs, sodium selenite and cyanobacterial extract.

The best synthesis of B-SeNPs was obtained by placing reaction by mixing 1 mM of 100 ml sodium selenite and 200 ml extract (1:2 ratio) for 24 h at 30 °C (Fig. 1a, b and c). The color change was not observed in control. However, the color changed from sky blue to white and finally to orange-red in the reaction mixture (Fig. 2a). The cyanobacteria derived selenium nanoparticles (B-SeNPs) were scanned for absorbance in the wavelength range from 200 to 700 nm. The maximum absorption peak was observed at 266 nm, this peak was not observed in the sodium selenite and extract. Sodium selenite did not give any absorption peak whereas, the B-SeNPs showed absorption peak at 266 nm and cyanobacterial aqueous extract showed the absorption peaks at 280 nm and 614 nm (Fig. 2b).

### GC–MS characterization of extract

**Figure 3:**
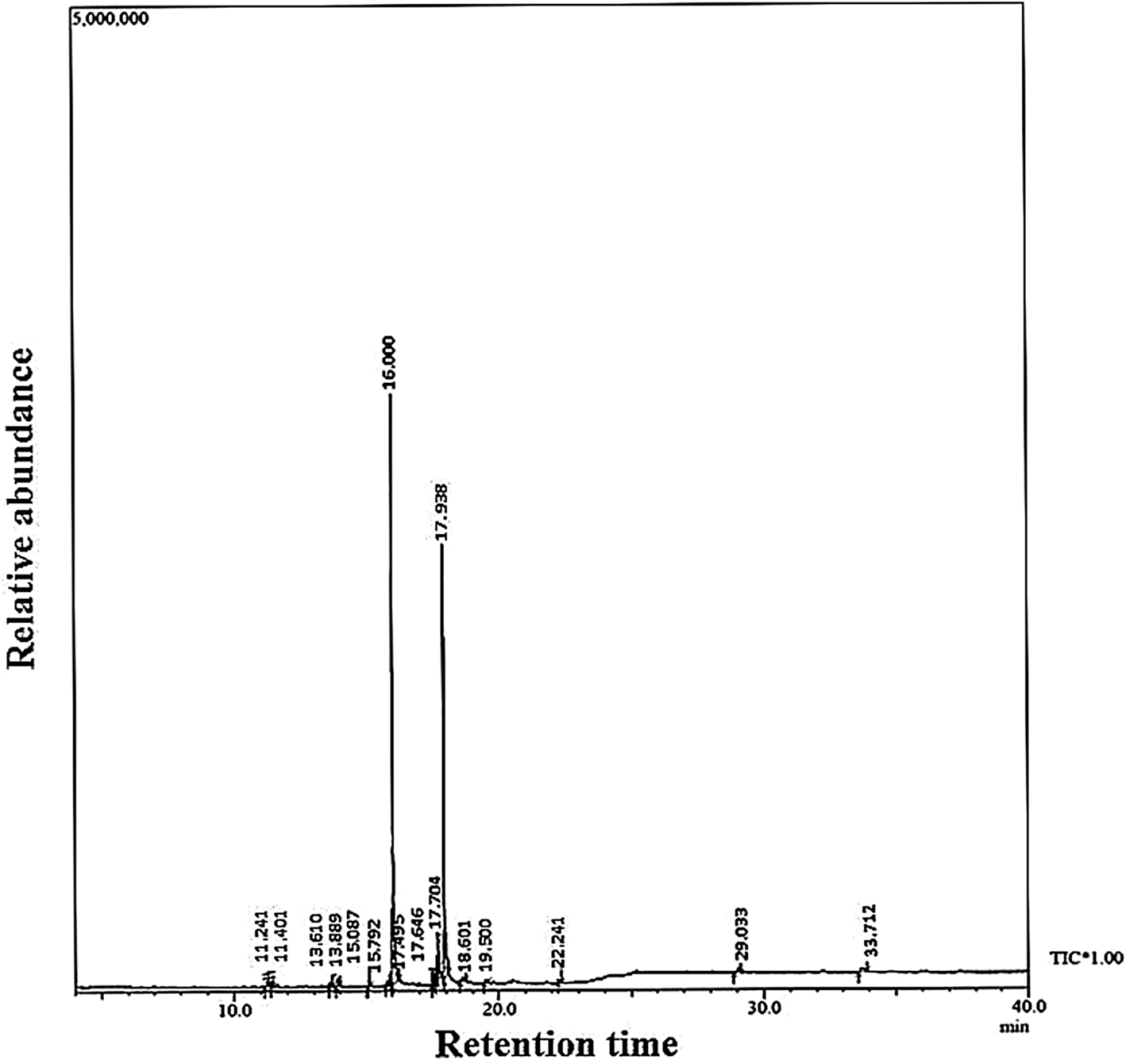
GC–MS profile of *Anabaena variabilis* cell extract.

Based on the retention time, mass spectra were identified with the help of NIST/Wiley Library. GC–MS profile of cyanobacterial extract showed 16 compounds-fatty acid esters (90.6%), carboxylic acid (1.42%), esters (1.64%), alkenes (2.85%), phenolics (0.39%), alkanes (0.40%), ethers (0.58%) and others (Fig. 3; Table 1).

**Table 1:** List of compounds found by GC–MS of cyanobacterial extract.

S. no	RT (min)	Area (%)	Compound	Other name	Nature
1	11.241	0.35	1,3-cyclohexadiene, 5-(1,5-dimethyl-4-hexenyl	Zingiberene	Cyclo-alkene derivative
2	11.401	0.39	Phenol, 3,5-bis(1,1-dimethylethyl)	Phenol, 3,5-di-tert-butyl-	Phenolic compound
3	13.610	0.40	Heptane, 3,3-dimethyl	3,3-Dimethylheptane	Alkane
4	13.889	0.84	Heptadecanoic acid, methyl ester	Methyl ester margaric acid methyl ester	Ester
5	15.087	1.24	Neophytadiene	2-(4,8,12-Trimethyltridecyl)buta-1,3-diene HL3QFB56FB	Alkene
6	15.792	0.38	9-octadecenoic acid (Z)-, methyl ester	Oleic acid, methyl ester	Ester
7	16.000	49.64	Hexadecanoic acid, methyl ester	Palmitic acid ester	Fatty acid ester
8	17.495	1.09	gamma.-Linolenic acid, methyl ester	cis-6,9,12-Octadecatrienoic acid, methyl ester	Fatty acid ester
9	17.646	1.41	9,12-Octadecadienoic acid, methyl ester	9,12-Octadecenoic acid, methyl ester	Fatty acid ester
10	17.704	2.13	(Z,Z)-6,9-CIS-3,4-Epoxy-nonadecadiene	-	Alkene
11	17.938	38.46	Methyl stearate	Stearic acid	Fatty acid ester
12	18.601	0.58	1,3-Propanediol, decyl ethyl ether	1-(3-ethoxypropoxy)tetradecane	Ether
13	19.500	0.42	Oxalic acid, 6-ethyloct-3-YL heptyl ester	2-O-(6-ethyloctan-3-yl) 1-O-heptyl oxalate	Ester
14	22.241	0.45	1,2-Benzenedicarboxylic acid	Phthalic acid	Dicarboxylic acid
15	29.033	0.97	2-(3-acetoxy-4,4,10,13,14-pentamethyl-2,3,4,5,6,7,10,11,12,13,14,15,16,17	Propanoic acid	Carboxylic acid
16	33.712	1.26	3-methyl-5-(2,6,6-trimethyl-1-cyclohexen-1-YL)-	3-Methyl-5-(2,6,6-trimethyl-1-cyclohexen-1-yl) isoxazole	Cyclo-alkene derivative

### Comparative FTIR analysis of B-SeNPs and extract

**Figure 4:**
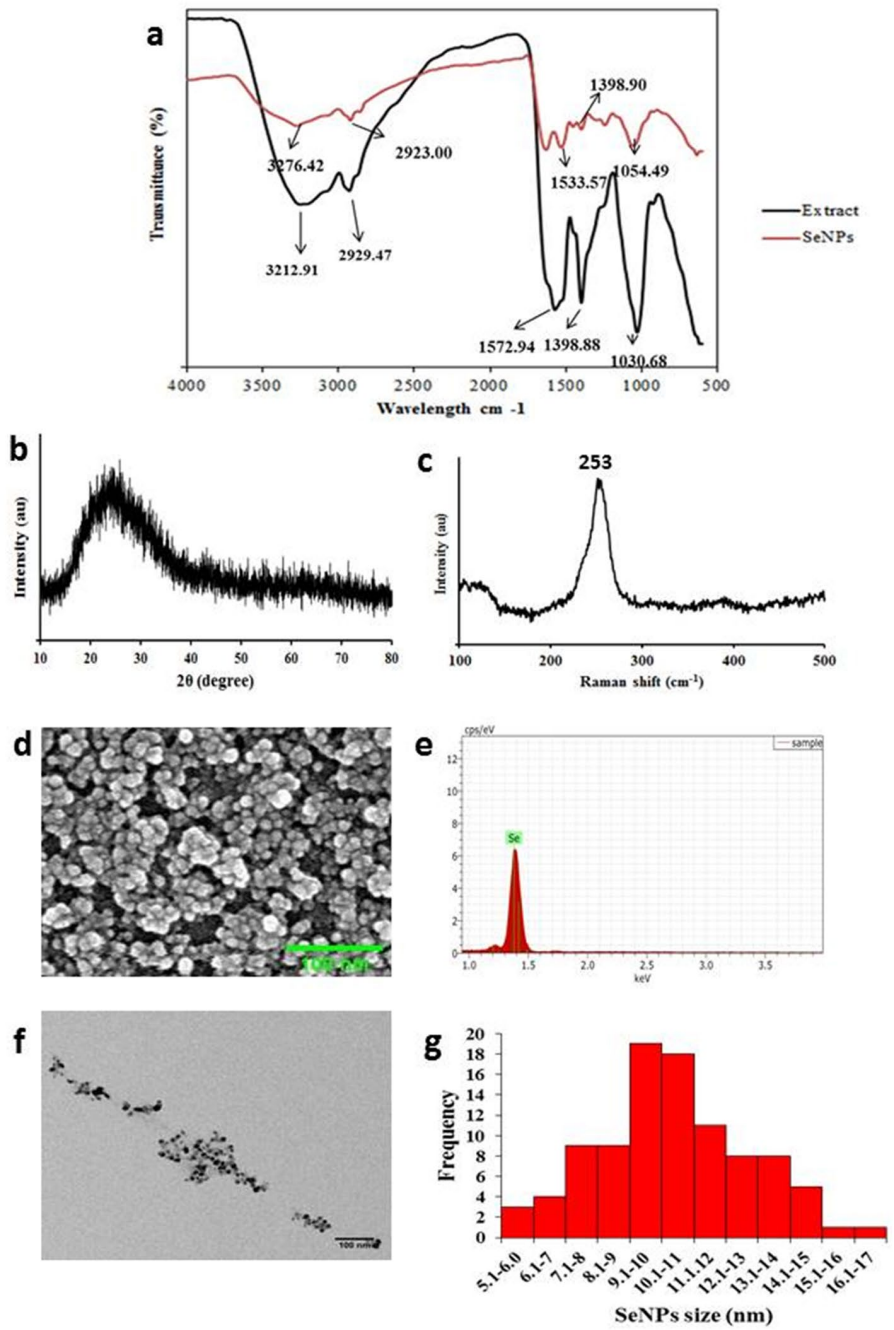
Physico-chemical characterization of B-SeNPs (**a**) FTIR spectra (**b**) XRD pattern (**c**) Raman spectra (**d**) SEM (**e**) EDX (**f**) TEM and (**g**) TEM Histogram for size distribution.

The peaks obtained in cyanobacterial extract and B-SeNPs represented functional groups in Fig. 4a and Table 2. The peak identification was done through past literature^31^.

**Table 2:** FTIR Spectra of cell extract and B-SeNPs.

S. no	Functional groups	Peak in Extract (cm^−1^)	Peak in SeNPs (cm^−1)^	References
1	Cyclohexane ring vibrations in saturated aliphatic group	1030.68	1054.49	^31^
2	OH bend in phenol or tertiary alcohol; COO^−^ symmetric stretching in fatty acids	1398.88	1398.98	^31,32^
3	Carboxylic group, CN stretching, NH bending	1572.94	1533.57	^31^
4	C-H stretch in alkanes	2929.47	2923.00	^31^
5	O–H bonded alcohols, phenols	3212.91	3276.42	^31^

### Characterization of B-SeNPs

SeNPs were characterized using X-ray diffraction analysis, Raman spectra, SEM–EDX and TEM. X-ray diffraction analysis was carried out to determine the phase of synthesized nanoparticles. Whole spectra of 2 h values, ranging from 20 θ to 80 θ were scanned. Orange-red B-SeNPs were amorphous in nature (Fig. 4b). Raman spectrum analysis of SeNPs derived from *Anabaena variabilis* NCCU-441 showed resonance peak at 253 cm^−1^ (Fig. 4c). Scanning electron microscopy-Energy dispersive X-ray spectroscopy (SEM–EDX) analysis of B-SeNPs exhibited 12 nm size of nanoparticles (Fig. 4d) with 94% weight of selenium. EDX spectrum gave a strong signal at 1.4 keV (Fig. 4e), which signified the presence of selenium. TEM analysis revealed that the synthesized cyanobacterial SeNPs were polymorphic, spherical and with an average size of 10.8 nm (Fig. 4f). 19 nanoparticles have the size range between 9–10 nm, 18 nanoparticles exhibited the size range between 10–11 nm and the overall range was found between 6.4–15.8 nm (Fig. 4g).

### Anti-oxidant activities of B-SeNPs and C-SeNPs

**Figure 5:**
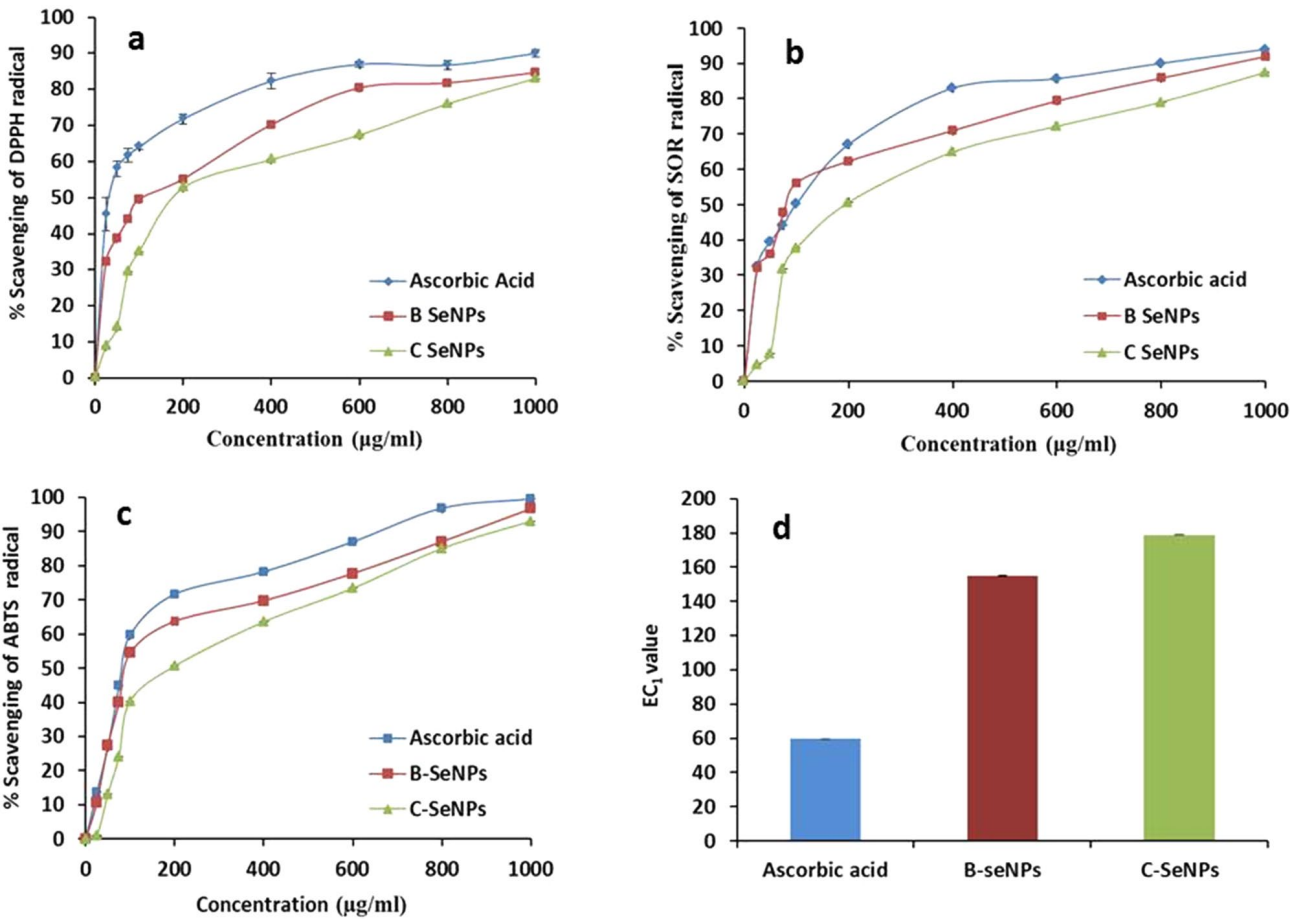
Comparative anti-oxidant assays of B-SeNPs and C-SeNPs (**a**) DPPH scavenging assay (**b**) SOR scavenging assay (**c**) ABTS assay (**d**) FRAP assay.

Anti-oxidant activities were performed by DPPH, FRAP, SOR and ABTS assays. Ascorbic acid was taken as the positive control. In DPPH, assay, IC_50_ obtained with B-SeNPs, C-SeNPs and ascorbic acid were 83.89 ± 2.11 μg/ml, 174.79 ± 0.29 and 56.36 ± 1.52 μg/ml. SOR assay showed that the B-SeNPs and C-SeNPs effectively scavenged the SOR free radicals with IC_50_ value of 80.55 ± 1.14 μg/ml and 176.84 ± 0.12 while ascorbic acid showed IC_50_ value of 74.95 ± 0.95 μg/ml. In ABTS assay, IC_50_ value was recorded as 92.58 ± 1.28 μg/ml, 239.11 ± 0.34and 84.71 ± 0.68 μg/ml for B-SeNPs, C-SeNPs and ascorbic acid respectively but the IC_50_ values for B-SeNPs were lower in comparison to C-SeNPs (significant at P ≤ 0.05). In FRAP assay, EC_1_ value of B-SeNPs, C-SeNPs and ascorbic acid were found to be 155.02 ± 0.93 µg/ml, 178.89 ± 1.84and 59.53 ± 0.53 µg/ml respectively (Table 3, Fig. 5a-d).

**Table 3:** Anti-oxidant activities of B-SeNPs and C-SeNPs.

Anti-oxidant assays	DPPH assay IC_50_ Value (μg/ml)	FRAP assay EC_1_ Value (μg/ml)	ABTS assay IC_50_Value (μg/ml)	SOR assay IC_50_ Value (μg/ml)
B-SeNPs	83.89 ± 2.11	155.02 ± 0.93	92.58 ± 1.28	80.55 ± 1.14
C-SeNPs	174.79 ± 0.29	178.89 ± 1.84	239.11 ± 0.34	176.84 ± 0.12
Ascorbic acid	56.36 ± 1.52	59.53 ± 0.53	84.71 ± 0.68	74.95 ± 0.95

### Anti-microbial activity of B-SeNPs and C-SeNPs

**Figure 6:**
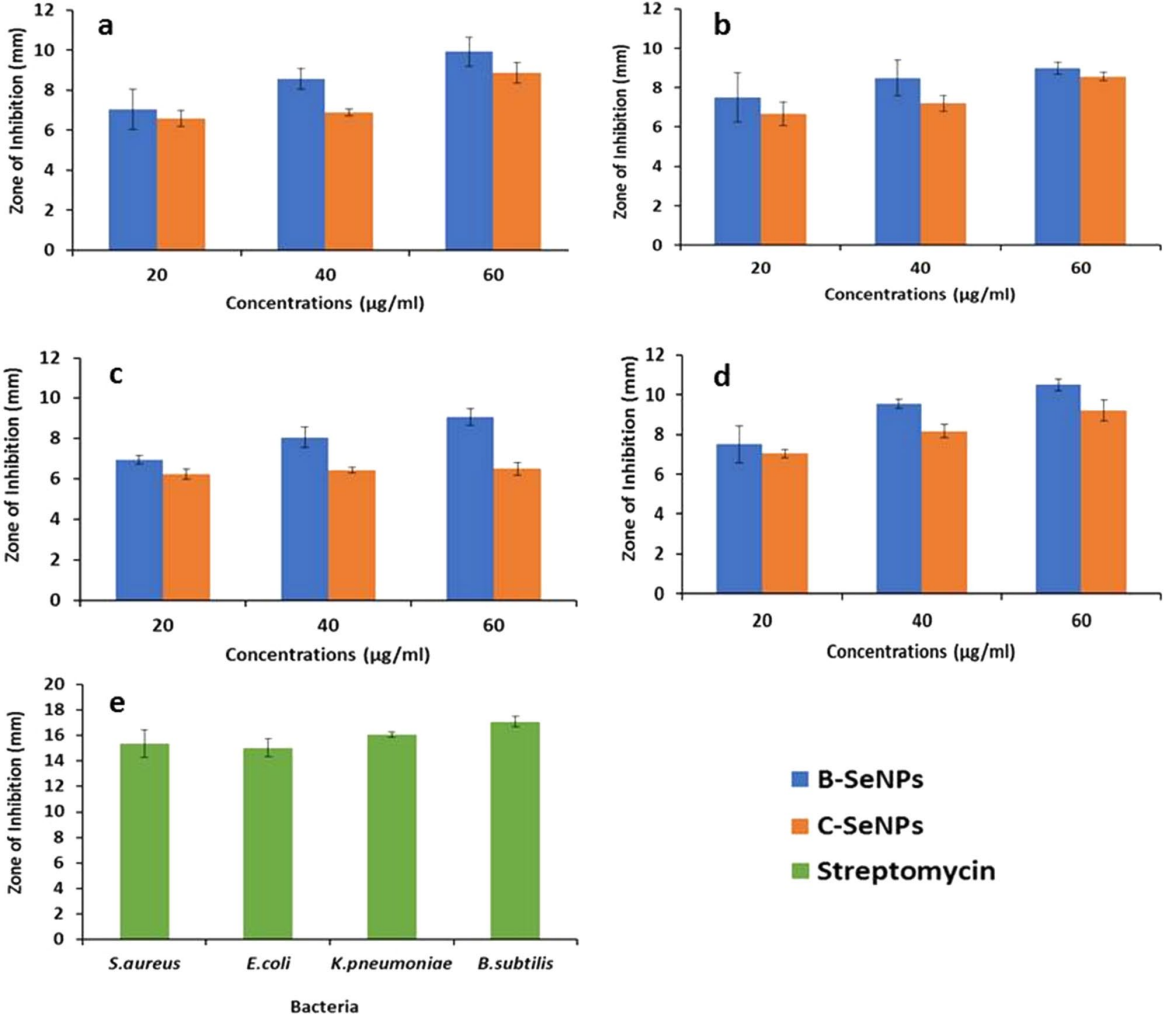
Comparative anti-bacterial activity of B-SeNPs and C-SeNPs by disc diffusion assay (**a**) *S. aureus* (**b**) *E. coli* (**c**) *K. pnuemonae* (**d**) *B. subtilis* (**e**) Streptomycin.

B-SeNPs and C-SeNPs showed the anti-bacterial activity against all the four tested bacterial strains at all the three concentrations 20, 40 and 60 µg/ml (Fig. 6a-e; Table 4). The zone of inhibition (ZOI) of B-SeNPs, C-SeNPs and streptomycin against all the bacterial strains have been mentioned in Table 4. Both B-SeNPs and C-SeNPs were found to be more efficient against the gram positive strain (*Bacillus subtilis*, *Staphylococcus aureus*) than the gram negative strains (*Klebsiella pneumonia*, *Escherichia coli*). The B-SeNPs were effective in comparison to C-SeNPs against all the tested bacterial strains due to the larger ZOI (significant at P ≤ 0.05).

**Table 4:** Anti-bacterial activity of B-SeNPs and C-SeNPs by disc diffusion method.

Concentrations (µg/ml)	Zone of inhibition (mm)							
	*S. aureus*		*E. coli*		*K. pneumoniae*		*B. subtilis*	
	B-SeNPs	C-SeNPs	B-SeNPs	C-SeNPs	B-SeNPs	C-SeNPs	B-SeNPs	C-SeNPs
20	7.0 ± 1	6.6 ± 0.4	7.5 ± 1.2	6.7 ± 0.6	7.0 ± 0.2	6.2 ± 0.2	7.5 ± 0.9	7.3 ± 0.2
40	8.5 ± 0.5	6.9 ± 0.2	8.5 ± 0.9	7.2 ± 0.4	8.0 ± 0.5	6.4 ± 0.2	9.5 ± 0.2	8.1 ± 0.4
60	10.0 ± 0.7	8.8 ± 0.5	9.0 ± 0.3	8.5 ± 0.2	9.0 ± 0.4	6.5 ± 0.3	10.5 ± 0.3	9.2 ± 0.5
Streptomycin	15.0 ± 1.1		15.0 ± 0.7		16.0 ± 0.2		17.0 ± 0.4	

**Figure 7:**
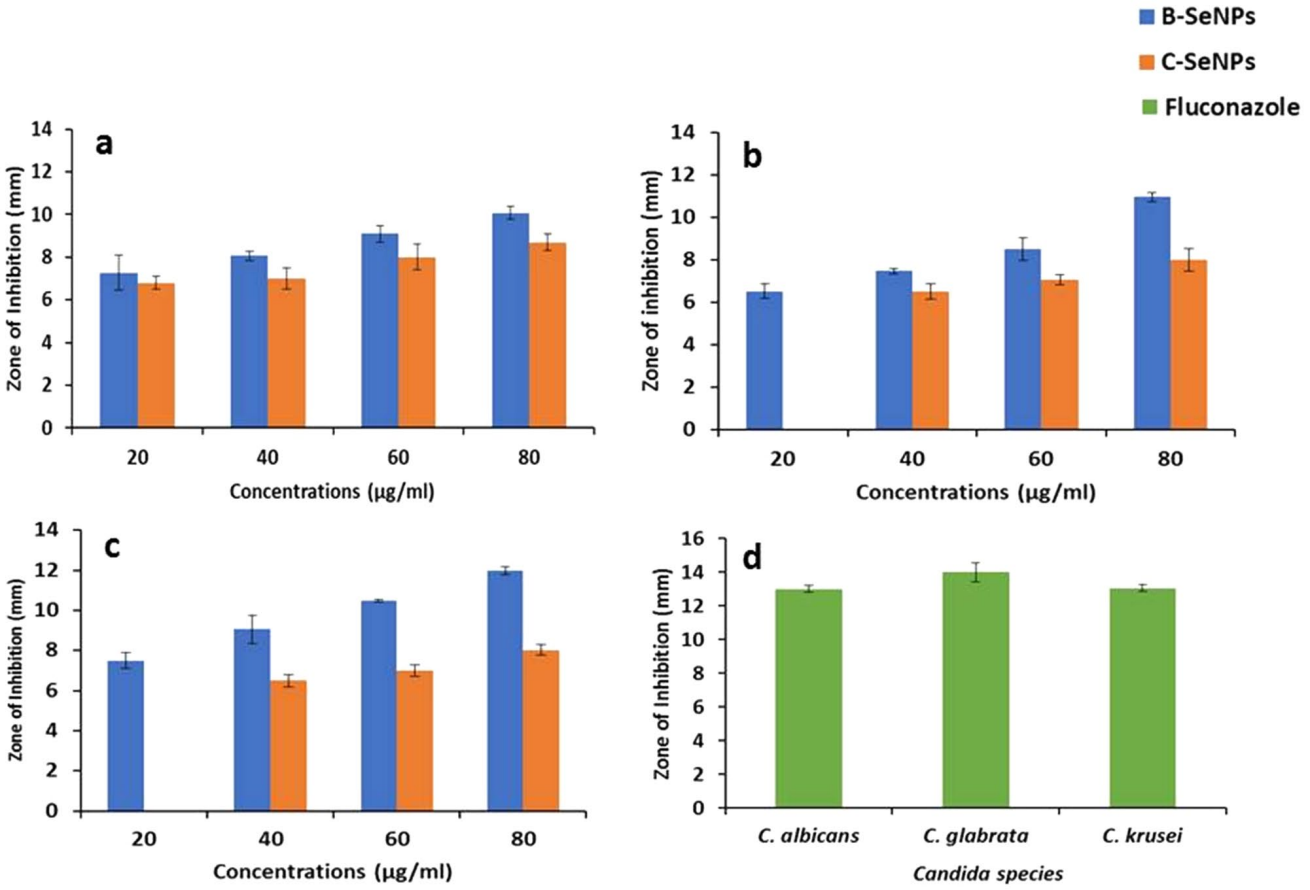
Comparative anti-fungal activity of B*-*SeNPs and C-SeNPs by disc diffusion assay. (**a**) *C. albicans* (**b**) *C. glabrata* (**c**) *C. krusei* (**d**) Fluconazole.

Anti-fungal activity of B-SeNPs and C-SeNPs were determined against the three species of *Candida* sp., viz. *Candida albicans*, *Candida glabrata* and *Candida krusei*. B-SeNPs gave larger zone of inhibition than C-SeNPs against all the three strains at all the concentrations (20, 40, 60 and 80 µg/ml) against *Candida krusei*. No ZOI could be observed in the least concentration (20 µg/ml) against *Candida glabrata* and *Candida krusei* in case of C-SeNPs (significant at P ≤ 0.05) (Fig. 7a-d; Table 5).

**Table 5:** Anti-fungal activity of B-SeNPs and C-SeNPs by disc diffusion method.

Concentrations (µg/ml)	Zone of inhibition (mm)					
	*Candida albicans*		*Candida glabrata*		*Candida krusei*	
	B-SeNPs	C-SeNPs	B-SeNPs	C-SeNPs	B-SeNPs	C-SeNPs
20	7.2 ± 0.8	6.8 ± 0.3	6.5 ± 0.3	No ZOI	7.5 ± 0.4	No ZOI
40	8.0 ± 0.2	7 ± 0.5	7.5 ± 0.6	6.5 ± 0.3	9.0 ± 0.7	6.5 ± 0.3
60	9.0 ± 0.4	8 ± 0.6	8.5 ± 0.5	7 ± 0.2	10.5 ± 0.6	7 ± 0.3
80	10.0 ± 0.3	8.7 ± 0.4	11.0 ± 0.2	8 ± 0.5	12.0 ± 0.2	8 ± 0.2
Fluconazole	13.0 ± 0.2		14.0 ± 0.5		13.0 ± 0.2	

### Anti-cancer activity of B-SeNPs and C-SeNPs

**Figure 8:**
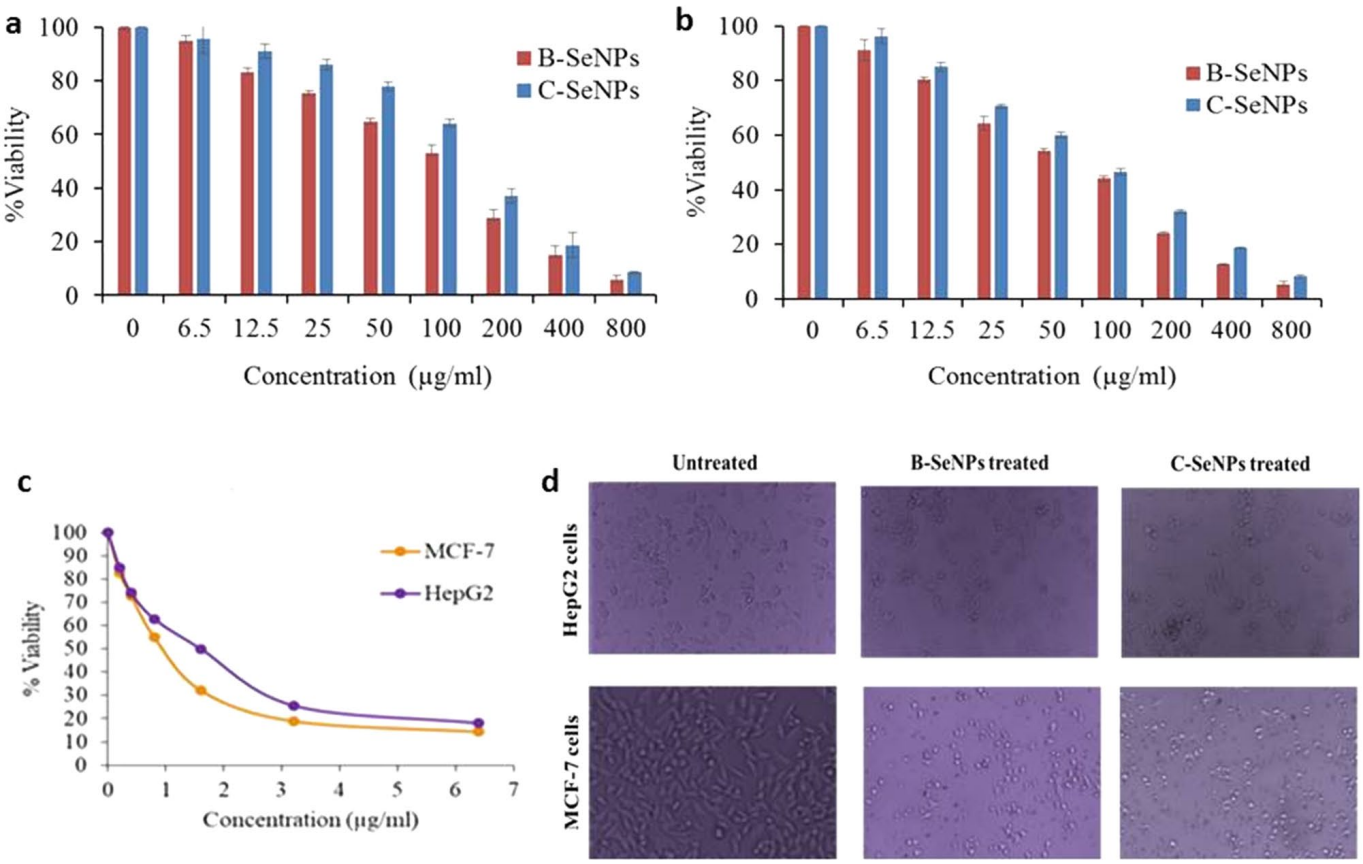
Comparative anti-cancer activity of B-SeNPs and C-SeNPs (**a**) MTT assay of HepG2 (**b**) MTT assay of MCF-7 (**c**) MTT assay of doxorubicin (**d**) Images before and after treatment of SeNPs for 24 h.

MTT assay was performed to find out the cytotoxicity effect of B-SeNPs and C-SeNPs on the cell lines (MCF-7 and HepG2). The results of B-SeNPs and C-SeNPs showed the concentration-dependent cytotoxic effects in both the cell lines after 24 h exposure (Fig. 8a-b, Table 6). The IC_50_ values for B-SeNPs were recorded as 49.69 ± 2.69 μg/ml and 96.22 ± 4.73 μg/ml. Whereas IC_50_ values of C-SeNPs were found to be 82.549 ± 1.65 μg/ml and 151.59 ± 4.06 μg/ml respectively. The IC_50_ values for standard drug (doxorubicin) was 0.81 ± 0.02 μg/ml and 1.80 ± 0.05 μg/ml against MCF-7 and HepG2 cells respectively (Fig. 8c, Table 6). These results showed that both types of SeNPs have anti-cancerous property but the B-SeNPs have better activity than C-SeNPs at lesser IC_50_ values (significant at P ≤ 0.05). The microscopic observation of B-SeNPs and C-SeNPs treated cells at their IC_50_ value showed considerable changes in the morphology of cells in comparison to untreated cells. The untreated cells appeared spindle shaped and closely adhered to each other, however the treated cells have fragmented and retracted morphology (Fig. 8d).

**Table 6:** Anti-cancer activity of B-SeNPs and C-SeNPs.

Cell lines	IC_50_ value (μg/ml)		
	B-SeNPs	C-SeNPs	Doxorubicin
MCF-7	49.69 ± 2.69	82.549 ± 1.65	0.81 ± 0.02
HepG2	96.22 ± 4.73	151.59 ± 4.06	1.80 ± 0.05

### Bio-compatibility assay of B-SeNPs and C-SeNPs on HEK-293 cell line

**Figure 9:**
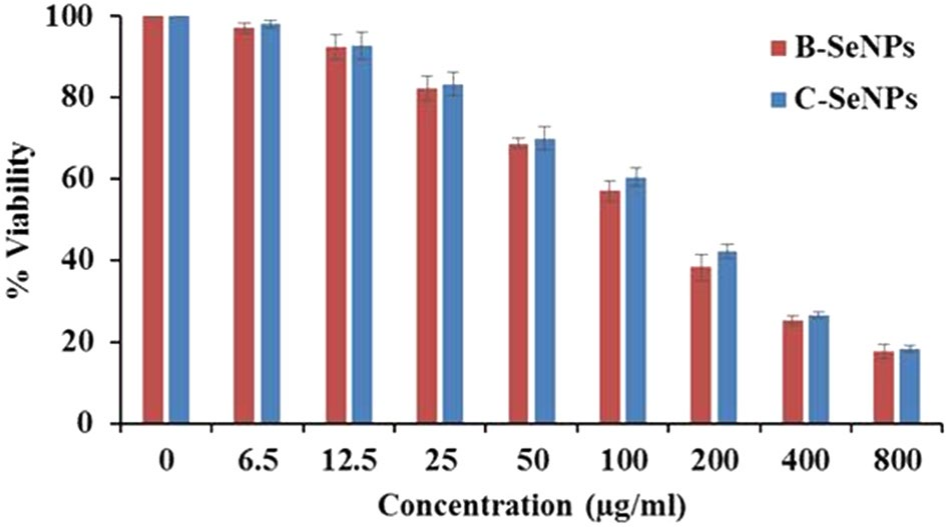
Biocompatibility assay of B-SeNPs and C-SeNPs on HEK-293 cell line.

The MTT assay was performed on normal cell line (HEK 293 cells) to check the bio-compatibility of B-SeNPs and C-SeNPs. The IC_50_ value of B-SeNPs and C-SeNPs against HEK-293 was found 143.21 ± 3.31 μg/ml and 154.69 ± 1.71 respectively (Fig. 9). The difference between IC_50_ value of normal cell and cancer cells of C-SeNPs were very close, that difference was much higher in case of B-SeNPs exposure than of C-SeNPs. So, the B-SeNPs were found to be non-toxic at their IC_50_ value and C-SeNPs were toxic toward normal cells (significant at P ≤ 0.05) (Fig. 9).

## Discussion

The biogenic SeNPs synthesis has got increased attention due to their wide applicability in biomedicine. The green synthesis method is preferred over physical and chemical methods as it is ecofriendly and does not depend on the use of harsh chemical and high energy^33^. For the very first time, cyanobacteria-*Anabaena variabilis* NCCU-441 has been used for the synthesis of SeNPs and comparison of their bioactivity with chemically synthesized commercial SeNPs. During the present investigation, B-SeNPs have been synthesized extra-cellularly using the crude cyanobacterial extract. Extra-cellular biogenic nanoparticles synthesis is much economical than intra-cellular synthesis as it saves chemicals, energy and time needed for cell disruption and purification. Use of cyanobacterial biomass have added advantage of their short life cycle.

The synthesis of B-SeNPs was visually observed as the color change of reaction mixture from sky blue to orange-red. Control showed no color change that shows their inability to synthesized SeNPs. B-SeNPs having orange red color have been also obtained in other species e.g. *Gliocladium roseum*^15^, *Allium sativum*^22^, *Emblica officinilis*^26^, *Pseudomonas putida* KT2440^34^, *Idiomarina* sp. PR58-8^35^. UV–Vis spectrophotometric scanning of B-SeNPs exhibited peak at 266 nm. The absorption maxima of extract at 280 nm may be due to the presence of proteins in the extract and at 614 nm is due to the presence of phycobilin pigments. Sky blue color of extract and absorption maxima of extract at 614 nm confirms the presence of phycocyanin in the extract^36^. The peak at 614 nm from the extract completely disappeared after the synthesis of B-SeNPs which may be due to their involvement and utilization in the capping of synthesized nanoparticles. When nanoparticle size is smaller than its Bohr excitation radius, the band gap gets enlarged due to the quantum confinement effect and this also influences nanomaterial band gap due to capping biomolecules and the proteins present on the surface. Other organisms have showed slightly different *λ*_max_ for SeNPs probably due to variation in their sizes e.g. at 330 nm in *Zooglea ramera*^14^, at 330 nm in *Gliocladium roseum*^15^, at 261 nm in *Bacillus megaterium*^17^, at 245 nm in *Aspergillus terrus*^19^, at 261 nm in *Diospyros montana*^21^, at 260 nm in *Allium sativum*^22^, at 381 nm in *Psidium guajava*^25^, at 271 nm in *Emblica officinalis*^26^ and at 395 nm in *Citrus limon*^27^.

During optimization, it was observed that the best synthesis occurred when extract and salt where mixed in a ratio of 2:1 for 24 h at 30 °C. B-SeNPs derived from other organisms have shown maximum synthesis at 30 °C e.g. bacteria-*Zooglea ramigera*^14^, fungi-*Gliocladium roseum*^15^ and plant-*Diospyros Montana*^21^. However, *Allium sativum* preferred 36 °C exposure for 5–7 days^22^. Moreover, *Aspergillus terreus*^19^ showed better synthesis at 25 °C and in lesser time (1 h). *Emblica officinilis* synthesized good SeNPs at 27 °C after 24 hours^26^.

GC–MS analysis of extract was done to know the probable biomolecules present in the cyanobacterial extract that may have helped in the synthesis of B-SeNPs. Two major peaks of fatty acid esters were observed i.e. Peak 1 with area 38.46% (Hexadecanoic acid, methyl ester) and Peak 2 with area 49.64% (Methyl stearate). These compounds have been also recorded in the GC–MS profile of *Streptomyces* sp.^37^ and *Mentha spicata*^38^ respectively. They have also suggested their reducing nature. Two more fatty acid esters, viz. ϒ-Linolenic acid, methyl ester and 9, 12-Octadecadienoic acid, methyl ester were found in lesser quantity (1.09% and 1.41% respectively). These fatty acid esters with reducing potential have been also found in *Allium saralicum*^39^ and *Citrus* wax^40^. Three esters (Heptadecanoic acid, methyl ester; 9-octadecenoic acid (Z)-, methyl ester; Oxalic acid, 6-ethyloct-3-YL heptyl ester) were also found in cyanobacterial extract. Heptadecanoic acid, methyl ester from *Mentha spicata*^31^, 9-octadecenoic acid (Z)-, methyl ester from *Thesium humile Vahl*^41^ and Oxalic acid, 6-ethyloct-3-YL heptyl ester from *Cakile maritima*^42^ were also reported for their good reducing potential. Apart from the esters, two carboxylic acids i.e. 1, 2-Benzenedicarboxylic acid and 2-(3-acetoxy-4, 4, 10, 13, 14-pentamethyl-) were also observed but in lesser amount. Some researchers have also reported the presence of these two carboxylic acids and reducing nature of cyanobacteria-*Calothrix brevisima*^43^ and angiosperm-*Punica species*^44^.

Compounds of other classes like alkane (Heptane, 3,3-dimethyl), alkene (Neophytadiene), cycloalkene derivatives (1,3-cyclohexadiene, 5-(1,5-dimethyl-4-hexenyl and 3-methyl-5-(2,6,6-trimethyl-1-cyclohexen-1-YL)-), phenol (Phenol, 3,5-bis(1,1-dimethylethyl) and ether (1,3-Propanediol, decyl ethyl ether) have been also obtained in the present study using *Anabaena variabilis* NCCU-441 extract. Previous study have also reported some related compounds like Heptane, 2,5,5-trimethyl-, a derivative of Heptane, 3,3-dimethyl from *Nostoc muscorum* NCCU-442^45^, Neophytadiene from *Plectranthus amboinicus*^46^, 1,3-cyclohexadiene, 5-(1,5-dimethyl-4-hexenyl from *Zingiber officinal*^47^, Phenol, 3,5-bis(1,1-dimethylethyl from *Calothrix brevisimma*^43^, 2 4 –Dimethylheptane, a derivative of 3,3-Dimethylheptane from *Pulicaria undulata*^48^, 1,3-Propanediol-2-ethyl-2-hydroxymethyl, a derivative of 1,3-Propanediol, decyl ethyl ether from *Lycium chinense* Miller fruits^49^ were also reported with good reducing ability. Thus, all these compounds from cyanobacterial extract are the probable reducing agents that reduce sodium selenite to B-SeNPs.

FTIR peaks in extract were present in SeNPs with slight shifting and reduced intensity. This indicates participation of these functional groups in the synthesis and capping of B-SeNPs. Cyclohexane ring vibrations in saturated aliphatic (alkane/alkyl) group were represented by the peaks at 1030.68 cm^−1^ and 1054.49 cm^−1^ in cell extract and SeNPs respectively. Peak at 1398.88 cm^−1^ represents OH bond in phenol or tertiary alcohol and COO^−^ symmetric stretching in the fatty acids in cell extract which was slightly different in SeNPs i.e. at 1398.98 cm^−1^^32^. Presence of carboxylic group, CN stretching and NH bending in amides was affirmed by the peak present at 1572.94 cm^−1^ (cell extract) and at 1533.57 cm^−1^ (B-SeNPs). C-H stretch in alkanes was found in both the cell extract and B-SeNPs as suggested by the peaks at 2929.47 cm^−1^ and 2923.00 cm^−1^ respectively. Further, peak at 3212.91 cm^−1^ (cell extract) and at 3276.42 cm^−1^ (B-SeNPs) represented O–H bonded alcohols and phenols^31^.

The presence of functional groups of alcohols, phenols, carboxylic acid, aromatic compounds, alkanes, alkenes and fatty acids in FTIR results perfectly correlates with the GC–MS results of extract where compounds having similar nature were observed. Ascorbic acid (a carboxylic acid) is a standard chemical reducing agent that has been used in many studies for the chemical and extracellular biogenic synthesis of SeNPs^29,50^. The chemical structure of ascorbic acid has three functional groups (alcohol, ester and alkene), their presence in the FTIR of B-SeNPs and cell extract suggest that they have played an important role in the synthesis and capping of B-SeNPs in present study also.

B-SeNPs were characterized by different techniques showed amorphous nature of SeNPs (XRD) having a size of 12 nm (SEM), 94% weight of selenium in EDX and having a prominent peak at 253 cm^−1^ in Raman spectrum with 10.8 nm size in TEM. Other biogenic SeNPs synthesized from *Spirulina platensis*^24^, *Bacillus* sp. MSh-1^1^ and *Pseudomonas aeruginosa* ATCC 27853^51^ as well as chemically synthesized also showed the amorphous XRD pattern^52^. Moreover, peak at 253 cm^−1^ is a characteristic absorption band for amorphous Se. *Spirulina* polysachcharides (SPS) based SeNPs have also shown a resonance peak at 253 cm^−1^^29^. SeNPs of different shapes and sizes have been also reported from different sources like from dried *Vitis vinifera* fruit (spherical, 3–18 nm)^24^, Arabic gum (spherical, 34 nm)^53^. Additionally, large sized spherical SeNPs have been also reported from *Spirulina* polysachcharides (90–550 nm)^29^ and *Capsicum annum* leaf (80 nm)^54^. *Aspergillus terreus* mediated SeNPs have also shown an average size of 47 nm with signal at 1.37 KeV^19^. *Gliocladium roseum* formed spherical SeNPs of 20–80 nm size with some larger particles (100–130 nm), that showed EDX signal at 1.4 KeV^15^. SeNPs synthesized from *Diospyros montana* also showed 94.44% of weight % with signal at 1.5 keV)^21^.

B-SeNPs and C-SeNPs were also tested and compared in the present study. B-SeNPs exhibited higher percentage of scavenging potential of free radicals by DPPH, SOR, APTS and FRAP. Higher percentage suggested the higher anti-oxidant potential. FRAP assay measures the anti-oxidant potential through the reduction of ferric ion (Fe^3+^) to ferrous ion (Fe^2+)^ by anti-oxidants.

To the best of our knowledge, there is no report of such comparative analysis. However, few information is available regarding biogenic SeNPs. *Diospyros montana* mediated SeNPs showed IC_50_ value of 0.225 μg/mL in DPPH scavenging assay^21^. *Emblica officinalis* mediated SeNPs showed dose-dependent free radical scavenging activity through DPPH and ABTS assays with EC_50_ values of 15.67 ± 1.41 and 18.84 ± 1.02 μg/mL respectively^27^. Menon et al., (2019) reported the IC_50_ value for *Zingiber officinale* based SeNPs as 125 μg/ml^23^. Cyanobacterial SeNPs gave significantly better activity in all the assays, so they could be considered as the better anti-oxidant than commercially synthesized SeNPs (significant at P ≤ 0.05).

Antimicrobial (anti-bacterial and anti-fungal) study showed that 60 µg/ml B-SeNPs led to the formation of an inhibition zone of 10 mm and 9 mm against *S. aureus* and *E. coli* respectively. In case of C-SeNPs, lesser diameter inhibition zone (8.8 and 8.5 respectively) were observed at the same concentration. Angiosperm*-Diospyros montana* mediated SeNPs showed inhibition zone of 8 mm and 7 mm against *S. aureus* and *E. coli* respectively^21^. *Psidium guajava* (angiosperm) based SeNPs showed even bigger zone of inhibition of (17 mm and 20 mm) against *E. coli* and *S. aureus* respectively^25^. Similarly, B-SeNPs showed larger zone of inhibition during anti-fungal activities than C-SeNPs in *Candida krucei*. But, overall better anti-fungal activity was found in B-SeNPs (significant at P ≤ 0.05). Anti-fungal activity of *Diospyros montana* mediated SeNPs was studied against *Aspergillus niger*. The assay was performed using disc diffusion method and the zone of inhibition was recorded as 12 mm. Further, the anti-fungal activity of e-waste mediated SeNPs has also been observed against *Candida albicans* and *Aspergillus niger*^55^. They found the minimum inhibitory concentration (MIC) as 6.5 μg/ml and 12.5 μg/ml respectively. It has been also observed that SeNPs synthesized by *Bacillus species* Msh-1 was active against *Aspergillus fumigatus* and *Candida albicans* with MIC values being 100 μg/ml and 70 μg/ml respectively^56^. B-SeNPs were found to be more effective on MCF-7 (IC_50_: 49.69) cells than HepG2 cells (IC_50_: 96.22) with the changed morphology. Also, C-SeNPs showed better anti-cancer activity in MCF-7 (IC_50_: 82.549) than HepG2 (IC_50_: 151.59) cells but the IC_50_ value was much lesser in B-SeNPs (significant at P ≤ 0.05). SeNPs synthesized from *Bacillus* sp. *MSh-1*^1^, *Diospyros montana*^21^, *Streptomyces bikiniensis strain Ess_amA-1*^51^ have also shown the IC_50_ value of 80.83 μg/ml, 41.5 μg/ml, 61.86 μg/mL against the MCF-7 (breast cancer) cell lines. SeNPs from *Emblica officinalis*^21^, *Streptomyces bikiniensis strain Ess_amA-1*^57^, *Withania somnifera*^58^exhibited IC_50_ value of 14.0 μg/ml, 25 μg/ml, 75.96 μg/ml (LD_50_) against A549 (lung cancer), N2a (brain cancer) and Hep-G2 (liver cancer) respectively.

Biocompatibility (anti-cancerous activity and low toxicity) can be considered as a remarkable property for any therapeutic agent directed towards the use of the human host. The results demonstrating the significant difference between the IC_50_ values of cancer cell line and normal cell line (significant at P ≤ 0.05) clearly put forward the fact that biosynthesized SeNPs have selectivity towards the cancerous cells as compared to normal cells^25^.

More potent bioactivities and less toxic nature of B-SeNPs than the C-SeNPs is may be due to the presence of different biomolecules on the surface of biosynthesized nanoparticles which enhanced their biomedical potential. Also, the synthesized B-SeNPs was of amorphous in nature and amorphous nanoparticles have better bioactivities than the crystalline nanoparticles^59^. Thus, the present study suggests that SeNPs synthesized using *Anabaena variabilis* NCCU-441 can be used as a therapeutic agent which are eco-friendly, cheap and non-toxic.

## Materials and methods

### Chemicals used

All cyanobacterial media chemicals (highly analytical grade) were procured from Merck except sodium selenite (Na_2_SeO_3_), Luria broth, Luria agar and yeast extract peptone dextrose (YPD) which was purchased from Himedia. Milli Q water was used for media preparation. Commercially synthesized SeNPs (average size of 50 nm) with ultra-high purity (99.9%) were procured from Nano Research Elements (CAS No 7782–49-2).

### Culture collection and maintenance

*Anabaena variabilis* NCCU-441 was procured from the Centre for Conservation and Utilisation of Blue Green Algae (CCUBGA), IARI, New Delhi, India and was cultured in BG-11 media (without sod. nitrate) under illumination of fluorescent light with 2000 ± 200 lx intensity for 12 h light and 12 h dark cycle. Biomass was harvested and thoroughly washed thrice with double distilled water, frozen and then lyophilized for further study.

### Extract preparation and its characterization

Lyophilized biomass powder was used to prepare 1 L aqueous extract by keeping it in water bath for 10 min at 60 °C. It was followed by centrifugation at 6000 rpm for 15 min at 4 °C and filtration by Whatman filter No.42. The chemical profile of cyanobacterial extract was analysed using GC–MS to find out the probable compounds that have reducing potential and aided the synthesis of nanoparticle. Samples for GC–MS analysis were prepared by dissolving the dried extract in methanol. The GC–MS analysis was done by Shimadzu GC–MS QP 2010 Plus equipment in electron ionization (EI) mode fitted with a RTX-5 capillary column (60 m × 0.25 mm × 0.25 μm). Helium was used as carrier gas with 0.7 ml min^–1^ of flow rate. The temperature of the injector was fixed at 260 °C. The initial and final temperature of the column was 80 °C and to 280 °C at the rate of 10 °C min^–1^ and 15 °C min^–1^ respectively. A 3.5 min solvent delay was used. Mass spectra were recorded under scan mode in the range of 40–650 m/z. Compounds were identified by comparing with NIST11/ WILEY library.

### Biogenic SeNPs synthesis and optimization

Color change of the reaction mixture was observed for the synthesis. Their UV–Vis spectra was scanned in between 200 to 700 nm. Sodium selenite and cell extract concentration ratio, reaction time and temperature were optimized for SeNPs synthesis. Tested ratio of sodium selenite (1 mM) and cell free extract (4 g/L) were 1:1, 1:2, 1:3 and 1:4. The best ratio of sodium selenite and cell free extract (1:2) of the reaction mixtures was then kept constant for determining effects of temperature (30 °C, 32 °C, 35 °C, 40 °C). After temperature optimization, reaction time was optimized. For this, the reaction was allowed for 6, 12, 18 and 24 h at optimized temperature (30 °C) and ratio (1:2). In all above conditions, reaction was allowed in the presence of fluorescent light with 2000 ± 200 lx intensity.

### Purification of B-SeNPs

Synthesized B-SeNPs were purified by centrifugation at 12,000 rpm for 20 min and repeated washing by double distilled water. The supernatant containing unreduced salt and unused capping agents were discarded while the pellet containing B-SeNPs was re-suspended in double distilled water and washed thrice. Finally, pellet was lyophilized at − 40 °C for physico-chemical and biological characterization.

### Physico-chemical characterization of B-SeNPs

FTIR was performed to determine the functional groups present on the surface of SeNPs. Powdered SeNPs were used and spectra were scanned between the range 600–3500 cm^−1^ by using TENSOR 37 FTIR Spectrometer. XRD was carried out to observe the diffraction pattern of synthesized SeNPs. For that, Lyophilized SeNPs were placed on glass slide and the diffraction pattern was studied by using Ultima IV X-ray diffractometer (Rigaku). The Raman spectroscopy was done at room temperature via Raman microscope (LabRAMHR800, HR800, JY). Freeze dried SeNPs were placed on a thin film and spectra were recorded in between the spectral range of 100–500 cm^−1^ with an interval of 10 s. SEM–EDX was performed to determine the shape, size, elemental composition and purity of synthesized nanoparticles. For that, SeNPs were dispersed in water and sonicated in Ultrasonicator (Thermotech PID-41 S). A drop of the sample was placed on carbon coated copper grid followed by coating of gold. The micrographs were captured on SEM (Nova NanoSEM 450), at accelerating voltage of 5 keV. The purity and elemental composition of synthesized SeNPs were obtained by EDX operated at 20 keV. The average size and range of synthesized nanoparticles was determined by performing TEM. SeNPs dispersed in water was placed on carbon coated grid and analyzed by using HRTEM-FEI 300 VHRTEM TECHNAI G2 30S TWIN. The size was calculated by using Image J software.

### Biological characterization

All the biological activities (anti-oxidant, anti-microbial, anti-cancer and biocompatibility) of B-SeNPs and C-SeNPs were determined for the comparative study.

### Determination of anti-oxidant activities of SeNPs by DPPH assay

2,2-diphenyl-1-picrylhydrazyl scavenging activity of B-SeNPs and C-SeNPs was determined by modified method of Brand-Williams^60^. 80% ethanol was used to prepare 0.1 mM DPPH. 3 ml DPPH and 1 ml SeNPs (B-SeNPs or C-SeNPs) having different concentrations (25 µg to 1000 µg) were mixed together and incubated in the dark for 30 min. Then, the absorbance was taken at 517 nm. Mixture of 3 ml DPPH and 1 ml of water was taken as a control and same procedure was followed for the control. % scavenging of SeNPs for DPPH was calculated by using the following formula:

1 $$\normalsize {{\% \ Scavenging \ of \ radical}} = \frac{{\left( {Ac - At} \right)}}{Ac} \times 100$$

where *Ac* = Absorbance of control, *At* = Absorbance of test.

Free radical scavenging activity of SeNPs was calculated from the percentage scavenging. IC_50_ values were calculated and compared with the standard-Ascorbic acid.

### Determination of anti-oxidant activities of SeNPs by SOR assay

According to Zhishen et al. (1999), for performing superoxide radical scavenging activity, 1 × 10^−2^ M methionine, 3 × 10^−6^ M Riboflavin, and 1 × 10^−4^ M nitroblue tetrazolium (NBT) (50 μM) were mixed together in 0.05 M of phosphate buffer (pH 7.8)^61^. 300 µl from the different concentrations of SeNPs (B-SeNPs or C-SeNPs) and 3 ml of the above mixture were mixed together and incubated in light for 30 min. One more similar set was kept in dark for the same time as blank. Absorbance was taken at 560 nm. % scavenging was calculated by adopting Eq. (1).

### Determination of anti-oxidant activities of SeNPs by FRAP assay

Ferric reducing anti-oxidant power assay was performed as described by Benzie and strain (1999)^62^. The FRAP reagent was prepared by adding 10 mM 2,4,6-Tri(2-pyridyl)-s-triazine (TPTZ) in a mixture of HCl (40 mM), FeCl_3_.6H_2_O (20 mM) and acetate buffer (300 Mm, pH-3.6) in 1:1:10 ratio. In 200 µl of SeNPs (B-SeNPs or C-SeNPs) 1.5 ml of FRAP reagent was added. The mixture was then incubated in dark at room temperature for 30 min. The absorbance was then measured at 593 nm using FRAP reagent as blank. Solutions of known Fe (II) concentrations (FeSO_4_.7H_2_O) were used for the preparation of the calibration curve. The parameter equivalent concentration (EC_1_) was defined as the concentration of anti-oxidant having a ferric-TPTZ reducing ability equivalent to that of 1 mM FeSO_4_.7H_2_O.

### Determination of anti-oxidant activities of SeNPs by ABTS radical cation scavenging assay

2,2'-azino-bis(3-ethylbenzothiazoline-6-sulfonic acid) (ABTS) activity of SeNPs was measured by using an improved method given by Re. et al., 2019^63^. ABTS aqueous solution (7 mM) was reacted with potassium persulfate (2.4 mM) in dark for 12–16 h at room temperature to produce stable ABTS radicals. Prior to assay, this solution was diluted in ethanol and equilibrated at 300 °C to give an absorbance of 0.70 ± 0.02 at 734 nm. To 10 µL of different concentration of SeNPs (B-SeNPs or C-SeNPs), 1 ml ABTS solution was added. Absorbance was measured at 734 nm against the blank (ethanol) at 30 °C exactly 30 min after the initial mixing and the percentage inhibition was calculated according to Eq. (1).

### Determination of anti-microbial activity of SeNPs by disc diffusion assay

Anti-bacterial activity of B-SeNPs or C-SeNPs was tested against gram positive (*Bacillus subtilis*, *Staphylococcus aureus*) and gram negative *(Escherichia coli* and *(Klebsiella pneumoniae)* bacteria through disc diffusion method. Overnight cultures were prepared by inoculating single colony in Luria broth separately. Obtained culture was set to 0.5 McFarland standards. 100 µl of revived culture was transferred to Mueller Hinton Agar (MHA) media plates and spread evenly. Discs having 6 mm diameter were saturated with 20, 40 and 60 µg biosynthesized SeNPs and placed over MHA media plates with inoculated bacterial culture following 24 h incubation at 37 °C to obtain zone of inhibition^64^. All the experiments were performed in triplicates and standard deviation was calculated. *Candida albicans*, *Candida krusei and Candida glabrata* were grown on yeast extract peptone dextrose (YPD) medium containing 2% glucose, 2% peptone, and 1% yeast extract (HiMedia, India) at 37 °C and 200 rpm. Approximately 10^5^ cells/ml were inoculated in molten agar (2%) media at 40 °C and poured into 100 mm diameter petri plates. 6 mm filter disks were kept on solid agar. Different concentrations of both types of SeNPs (20, 40, 60 and 80 µg) were suspended in distilled water and pipetted onto filter disks. Fluconazole (4 μg/disk) was used as a positive control. The diameter of the zone of inhibition was recorded in millimeters after 24 h and was compared with control^65^.

### Determination of anti-cancer activity of SeNPs by MTT assay

3-(4,5-dimethylthiazol-2-yl)-2, 5-diphenyltetrazolium bromide **(**MTT) assay was performed to check cytotoxicity of SeNPs (B-SeNPs or C-SeNPs) using the method of Mossman (1983)^66^. 1 × 10^4^ mL^−1^ cell lines (MCF-7 and HepG2) in their exponential growth phase were grown in a 96-well plate, then incubated in a CO_2_ incubator at 37 °C for 24 h. Different concentrations of B-SeNPs/C-SeNPs (0, 6.5, 12.5, 25, 50,100, 200, 400 and 800 μg/ml) was then added to the plate in triplicates. After 24 h of incubation, 20 μL MTT reagent was added in each well and incubated, formazan crystals were formed after 4 h of incubation. After adding 150 μL of detergent in each well, plates were read in a microplate reader at 570 nm (BIO-RAD microplate reader-550). Untreated and 24 h treated cell lines by 0, 6.5, 12.5, 25, 50,100, 200, 400 and 800 μg/ml of SeNPs for 24 h were used for cell viability test. Images were captured before and after the treatment. Doxorubicin was used as the standard drug. Percentage viability was calculated using equation:

$${{\% \ Viability}} = \frac{{\left( {Ac - At} \right)}}{Ac} \times 100$$

where *Ac* = Absorbance of control, *At* = Absorbance of test.

### Bio-compatibility assay of SeNPs on HEK-293 cell line

MTT assay was also performed to check the compatibility of B-SeNPs and C-SeNPs towards HEK 293 cells (normal kidney embryonic cell line)^25^.

### Statistical analysis

Data were presented as mean ± SD. Statistical analysis was performed by one way ANOVA followed by least significant difference at the level of 95% significance using Graph Pad software 8.1, San Diego, California.

## Concluding remarks

The present study highlights the *Anabaena variabilis* NCCU-441 cell extract mediated synthesis of B-SeNPs, its optimization and its comparative bioactivities with the commercial SeNPs. Orange-red colored, small (10.8 nm), spherical shaped SeNPs were successfully synthesized which were of high purity (94%). B-SeNPs showed better anti-oxidant, anti-microbial and anti-cancer activity than C-SeNPs. Thus, cyanobacteria mediated synthesis can be considered as safe and non-toxic way to synthesize SeNPs, that can be used as a probable drug candidate against cancer, microbial disease etc.

## Supplementary Information

Supplementary Information.
