# Supplementary figures and images for: Biomedical potential of Anabaena variabilis NCCU-441 based Selenium nanoparticles and their comparison with commercial nanoparticles
Source: Sci Rep. 2021 Jun 29;11:13507. doi: 10.1038/s41598-021-91738-7 (PMC8242014; doi:10.1038/s41598-021-91738-7)

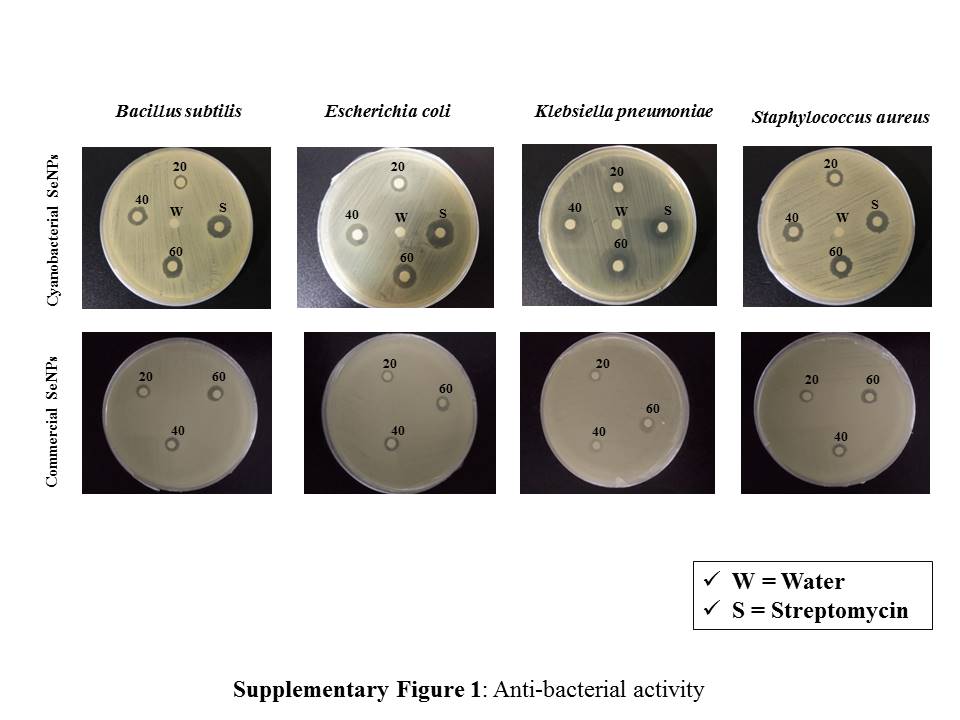


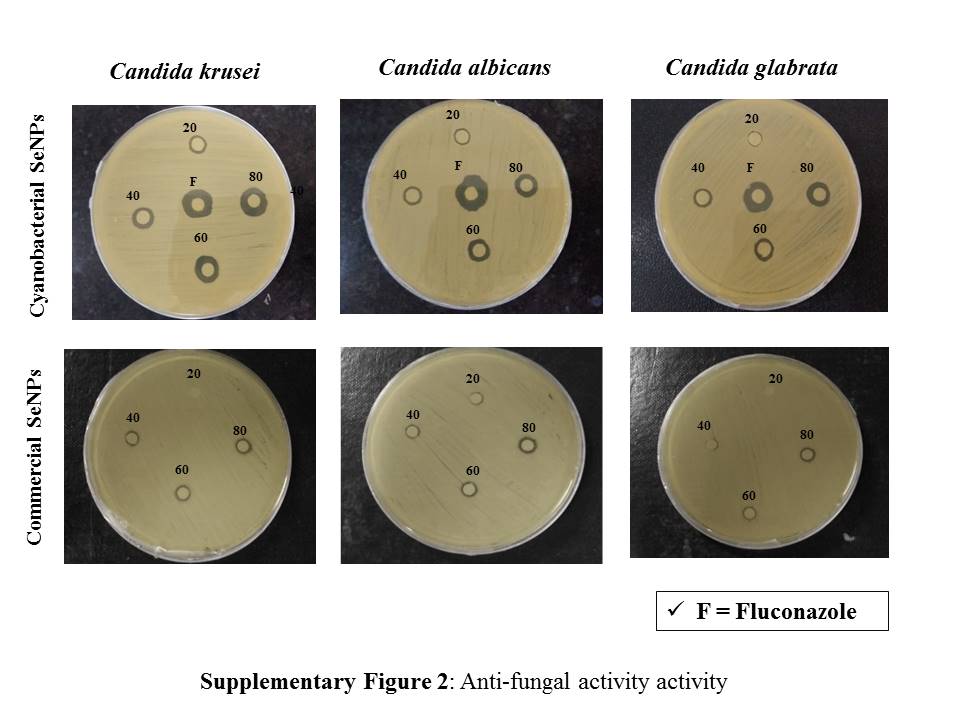

Supplement: Supplementary file 1 — Supplementary Information. [file 41598_2021_91738_MOESM1_ESM.docx]
